# Supplementary material for: The Small RNA Universe of Capitella teleta
Source: Front Mol Biosci. 2022 Feb 25;9:802814. doi: 10.3389/fmolb.2022.802814 (PMC8915122; doi:10.3389/fmolb.2022.802814)
Supplement: Supplementary file 1 [file DataSheet1.ZIP › Supplement/known/CAPTEscaffold_39_4065.pdf]

Provisional ID	:	CAPTEscaffold_39_4065
Score total	:	213466.4
Score for star read(s)	:	3.9
Score for read counts	:	213456.3
Score for mfe	:	1.7
Score for randfold	:	1.6
Score for cons. seed	:	3
Total read count	:	418697
Mature read count	:	417662
Loop read count	:	47
Star read count	:	988

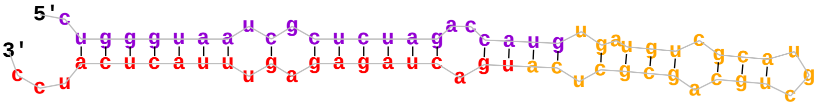

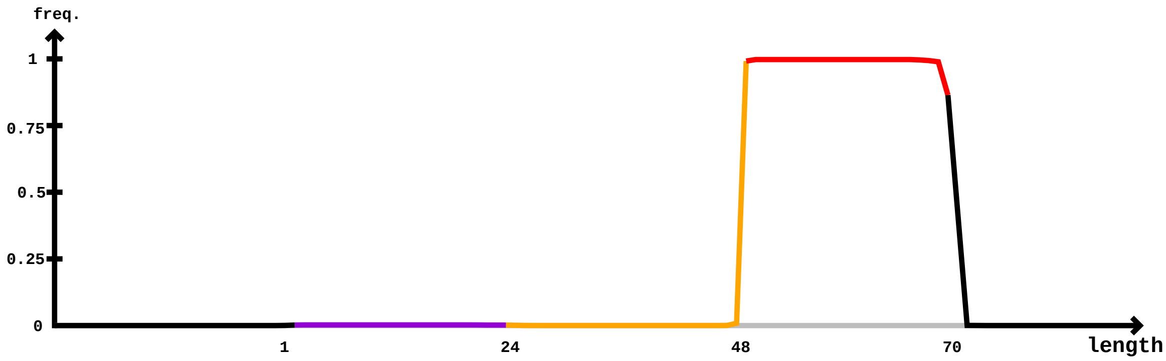

Star   Mature

```
5'- uucucugcugacugccugucugucuggguaaucgcucuagaccaugugaugucgcaugcugcagcgcucaugacuagagaguuuacucauccagagaguagucuccuucuu  -3'  obs
    uucucugcugacugccugucugucuggguaaucgcucuagaccaugugaugucgcaugcugcagcgcucaugacuagagaguuuacucauccagagaguagucuccuucuu       exp
    uucucugcugacugccugucugucuggguaaucgcucuagaccaugugaugucgcaugcugcagcgcucaugacuagagaguuuacucauccagagaguagucuccuucuu       known
    .........((((((.(.((((..(((((((.(.((((((..((((.(.(((.(((...))).)))).)))).)))))).).)))))))..)))).)))))))........   reads  mm  sample
    ......................ucuggguaaucgcucuaga......................................................................   3      0   seq
    ......................ucuggguaaucgcucuagac.....................................................................   3      0   seq
    ......................ucuggguaaucgcucuagacc....................................................................   135    0   seq
    ......................Acuggguaaucgcucuagacc....................................................................   1      1   seq
    ......................ucuAgguaaucgcucuagacc....................................................................   1      1   seq
    ......................ucuggguaaucgGucuagacc....................................................................   1      1   seq
    ......................ucuggguaaucgcucuagCcc....................................................................   1      1   seq
    ......................ucAggguaaucgcucuagacc....................................................................   1      1   seq
    ......................ucuggguaaucgcucuagaccau..................................................................   20     0   seq
    ......................ucuggguaaucgcucuagaccauU.................................................................   2      1   seq
    ......................ucuggCuaaucgcucuagaccaug.................................................................   1      1   seq
    ......................ucuggguaaucgcucuagaccaug.................................................................   28     0   seq
    .......................cuggguaaucgcucuagac.....................................................................   6      0   seq
    .......................cuggguaaucgcucuagacc....................................................................   25     0   seq
    .......................cuggguaaucgcucuagacca...................................................................   4      0   seq
    .......................cugggAaaucgcucuagaccau..................................................................   1      1   seq
    .......................cuggguaaucgcucuagaccau..................................................................   15     0   seq
    .......................cuggguaaucgcucuagaccauU.................................................................   3      1   seq
    .......................cuggguaaucgcucuagacAaug.................................................................   1      1   seq
    .......................cuAgguaaucgcucuagaccaug.................................................................   1      1   seq
    .......................cuggguaaucgcucuagaccaug.................................................................   565    0   seq
    .......................cuggguaaucgcGcuagaccaug.................................................................   1      1   seq
    .......................cuggguaaucgcucuagacUaug.................................................................   2      1   seq
    .......................Uuggguaaucgcucuagaccaug.................................................................   1      1   seq
    .......................cuggguaaucgcucuagCccaug.................................................................   1      1   seq
    .......................cuggguaaucgcucuagaccaugu................................................................   5      0   seq
    .......................cuggguaaucgcucuagaccaugA................................................................   1      1   seq
    .......................cuggguaaucgcucuagaccaugC................................................................   1      1   seq
    .......................cuggguaaucgcucuagaccauguU...............................................................   1      1   seq
    ........................uggguaaucgcucuagacc....................................................................   1      0   seq
    ........................uggguaaucgcucuagacca...................................................................   3      0   seq
    ........................uggguaaucgcucuagaccau..................................................................   1      0   seq
```

Star **Mature**

```
uucucugcugacugccugucuguCUGGGUAAUCGCUCUAGACCAUGugaugucgcaugcugcagcgcucaugacuagagaguuuacucauccagagaguagucuccuucuu
```

```
uucucugcugacugccugucugucuggguaaucgcucuagaccaugugaugucgcaugcugcagcgcucaugacuagagaguuuacucauccagagaguagucuccuucuu
........................uggguaaucgcucuagaccaug.................................................................    14    0    seq
........................uggguaaucgcucuagaccaugu................................................................    55    0    seq
........................uggguaaucgcucuagaccaugug...............................................................    62    0    seq
........................uggguaaucgcucuagaccauguga..............................................................    17    0    seq
........................uggguaaucgcucuagaccaugugG...............................................................    1     1    seq
........................uggguaaucgcucuagaccaugugU...............................................................    2     1    seq
.........................ggguaaucgcucuagacc....................................................................    1     0    seq
..............................................ugaugucgcaugcugcagcg.............................................    3     0    seq
..............................................ugaugucgcaugcugcagcgc............................................    2     0    seq
..............................................ugaugucgcaugcugcagcgcu...........................................    7     0    seq
..............................................ugaugucgcaugcugcagcgcuc..........................................    11    0    seq
..............................................ugaugucgcaugcugcagcgcuca.........................................    24    0    seq
....................................................................caugacuagagaguuuacuc.......................    2     0    seq
....................................................................caugacuagagaguuuacuca......................    5     0    seq
....................................................................caugacuagagaguuuacucC......................    1     1    seq
....................................................................caugacuagagaguuuacucau.....................    9     0    seq
....................................................................caugacuagagaguuuacucauA....................    1     1    seq
....................................................................caugacuagagaguuuacucauc....................    101   0    seq
....................................................................caugacuagagaguuuacucaucU...................    1     1    seq
....................................................................caugacuagagaguuuacucaucca..................    1     0    seq
.....................................................................augacuagagaguuuacu........................    4     0    seq
.....................................................................augacuagagaguuuacuc.......................    4     0    seq
.....................................................................Cugacuagagaguuuacuc.......................    1     1    seq
.....................................................................augacuagagaguuuacuca......................    17    0    seq
.....................................................................augacuagagaguuuacucau.....................    98    0    seq
.....................................................................Cugacuagagaguuuacucauc....................    29    1    seq
.....................................................................augacuagagaguuuacucGuc....................    3     1    seq
.....................................................................augacuagagaguAuacucauc....................    3     1    seq
.....................................................................augacuagagaguuuacucaAc....................    2     1    seq
.....................................................................augacuagagaguuCacucauc....................    2     1    seq
.....................................................................augacuGgagaguuuacucauc....................    4     1    seq
.....................................................................augacuagagaguuuacCcauc....................    1     1    seq
.....................................................................augacuagUgaguuuacucauc....................    1     1    seq
.....................................................................augacuagagaguuuacucauU....................    3     1    seq
.....................................................................augaAuagagaguuuacucauc....................    1     1    seq
.....................................................................augacuagagaguuuacucauA....................    1     1    seq
.....................................................................augacuagagaguGuacucauc....................    1     1    seq
.....................................................................augacuagagagAuuacucauc....................    1     1    seq
.....................................................................augacuagagaguuuacAcauc....................    2     1    seq
.....................................................................augacuagagaguuuacucauG....................    1     1    seq
.....................................................................augacuagagaguuuGcucauc....................    1     1    seq
.....................................................................aAgacuagagaguuuacucauc....................    1     1    seq
.....................................................................augacuagagaguuuacucauc....................    2580  0    seq
.....................................................................augacAagagaguuuacucauc....................    4     1    seq
.....................................................................Gugacuagagaguuuacucauc....................    1     1    seq
.....................................................................augacuagaUaguuuacucauc....................    1     1    seq
.....................................................................augacuagagaguuuacucaucA...................    4     1    seq
.....................................................................augacuagagaguuuacucaucU...................    14    1    seq
.....................................................................augacuagagaguuuacucaucc...................    38    0    seq
.....................................................................Cugacuagagaguuuacucaucc...................    577   1    seq
.....................................................................augacuagagaguuuacucaucAa..................    1     1    seq
.....................................................................augacuagagaguuuacucauccU..................    3     1    seq
......................................................................ugacuagagaguuuacuc.......................    524   0    seq
......................................................................Agacuagagaguuuacuc.......................    1     1    seq
......................................................................ugacuagagaguuuacAc.......................    1     1    seq
......................................................................ugacuagagaguuuacuca......................    1024  0    seq
......................................................................Cgacuagagaguuuacuca......................    1     1    seq
......................................................................ugaUuagagaguuuacuca......................    1     1    seq
......................................................................ugacuagagaguuuacuAa......................    1     1    seq
......................................................................ugacuagagaguAuacuca......................    1     1    seq
......................................................................ugacuagagaguuuacucC......................    1     1    seq
......................................................................Agacuagagaguuuacuca......................    3     1    seq
......................................................................ugacAagagaguuuacuca......................    1     1    seq
......................................................................ugacCagagaguuuacuca......................    1     1    seq
......................................................................ugCcuagagaguuuacucau.....................    2     1    seq
......................................................................ugacAagagaguuuacucau.....................    1     1    seq
......................................................................ugacuagagaguuuacucau.....................    1812  0    seq
......................................................................ugacuagagaguuuacuUau.....................    2     1    seq
......................................................................ugacuagagagAuuacucau.....................    1     1    seq
......................................................................Cgacuagagaguuuacucau.....................    1     1    seq
```

Star   Mature

uucucugcugacugccugucugucuggguaaucgcucuagaccaugugaugucgcaugcugcagcgcucaugacuagagaguuuacucauccagagaguagucuccuucuu			
....ugacuagagaguuuacucGu.....................	1	1	seq
....ugacuagagaguuuacucaC.....................	1	1	seq
....ugacuagUgaguuuacucau.....................	1	1	seq
....ugacuagaAaguuuacucau.....................	1	1	seq
....Agacuagagaguuuacucau.....................	8	1	seq
....ugacuagagaguuuacucaA.....................	2	1	seq
....ugacuagagUguuuacucau.....................	1	1	seq
....Ggacuagagaguuuacucau.....................	2	1	seq
....uAacuagagaguuuacucau.....................	2	1	seq
....ugacuagagaguCuacucau.....................	1	1	seq
....ugUcuagagaguuuacucau.....................	1	1	seq
....ugaUuagagaguuuacucauc....................	2	1	seq
....ugacuagagaguuuacucauU....................	19	1	seq
....Cgacuagagaguuuacucauc....................	3	1	seq
....ugacuagagagCuuacucauc....................	5	1	seq
....ugacuagagaguuuacucaAc....................	16	1	seq
....ugaAuagagaguuuacucauc....................	7	1	seq
....ugacuagagaguuuacuUauc....................	14	1	seq
....ugacuagagaguuGacucauc....................	1	1	seq
....ugacuagaUaguuuacucauc....................	1	1	seq
....ugacuagagaUuuuacucauc....................	1	1	seq
....ugacuagagaguuuacucauA....................	52	1	seq
....ugacuagagaguuuacucaCc....................	3	1	seq
....ugacuagagaguuuacucGuc....................	7	1	seq
....uCacuagagaguuuacucauc....................	7	1	seq
....ugacuagagaguCuacucauc....................	3	1	seq
....ugacuagagaguuuacucNuc....................	1	1	seq
....ugacAagagaguuuacucauc....................	42	1	seq
....Agacuagagaguuuacucauc....................	215	1	seq
....ugacuagUgaguuuacucauc....................	7	1	seq
....ugacuagagaguuuaAucauc....................	6	1	seq
....ugacuagagaguuuaUucauc....................	8	1	seq
....uAacuagagaguuuacucauc....................	18	1	seq
....ugacuagagaguuuacuGauc....................	4	1	seq
....ugacuagagaguGuacucauc....................	5	1	seq
....ugacuagagaguuuGcucauc....................	13	1	seq
....ugacuagagaguuuacucauc....................	48635	0	seq
....ugacuagCgaguuuacucauc....................	1	1	seq
....ugacuagagaguuuUcucauc....................	6	1	seq
....ugacuagagGguuuacucauc....................	5	1	seq
....ugacuagaCuuuacucauc....................	1	1	seq
....ugacuagagaguuuacucauG....................	3	1	seq
....Ngacuagagaguuuacucauc....................	17	1	seq
....ugacuagagaguuuaGucauc....................	4	1	seq
....ugacuagaAaguuuacucauc....................	22	1	seq
....ugacuaUagaguuuacucauc....................	6	1	seq
....ugacuagagaguAuacucauc....................	9	1	seq
....ugCcuagagaguuuacucauc....................	1	1	seq
....ugacuUgagaguuuacucauc....................	5	1	seq
....ugacuGgagaguuuacucauc....................	16	1	seq
....ugUcuagagaguuuacucauc....................	7	1	seq
....ugacuagGgaguuuacucauc....................	4	1	seq
....ugacuagagUguuuacucauc....................	7	1	seq
....ugacuagagaguuuacuAauc....................	10	1	seq
....ugacGagagaguuuacucauc....................	2	1	seq
....ugacuagagaguuuacCcauc....................	4	1	seq
....ugacuagagaAuuuacucauc....................	1	1	seq
....ugGcuagagaguuuacucauc....................	5	1	seq
....ugacuagagaguuuacGcauc....................	1	1	seq
....ugacuagagaguuAacucauc....................	10	1	seq
....ugacCagagaguuuacucauc....................	7	1	seq
....uUacuagagaguuuacucauc....................	6	1	seq
....ugacuagagaguuuCcucauc....................	2	1	seq
....ugacuagagaguuuacucUuc....................	8	1	seq
....ugacuagagaguuuacAcauc....................	7	1	seq
....ugacuagaCaguuuacucauc....................	2	1	seq
....ugacuagagaguuCacucauc....................	15	1	seq
....ugacuaAagaguuuacucauc....................	13	1	seq
....ugacuagagagAuuacucauc....................	14	1	seq
....Ggacuagagaguuuacucauc....................	19	1	seq

```
                        Star                                           Mature
uucucugcugacugccugucugucuggguaaucgcucuagaccaugugaugucgcaugcugcagcgcucaugacuagagaguuuacucauccagagaguagucuccuucuu
......................................................................ugacuNgagaguuuacucauc....................   2       1   seq
......................................................................Cgacuagagaguuuacucaucc...................   54      1   seq
......................................................................ugacuagagaguuuGcucaucc...................   91      1   seq
......................................................................ugacuagagaCuuuacucaucc...................   2       1   seq
......................................................................ugacuagagaguuuacuGaucc...................   25      1   seq
......................................................................ugacuagagaAuuuacucaucc...................   19      1   seq
......................................................................ugacuagGgaguuuacucaucc...................   48      1   seq
......................................................................ugacuaUagaguuuacucaucc...................   23      1   seq
......................................................................ugacuagagaUuuuacucaucc...................   16      1   seq
......................................................................ugacuagagaguuAacucaucc...................   36      1   seq
......................................................................ugacAagagaguuuacucaucc...................   261     1   seq
......................................................................ugCcuagagaguuuacucaucc...................   3       1   seq
......................................................................ugNcuagagaguuuacucaucc...................   3       1   seq
......................................................................ugacuagaCaguuuacucaucc...................   8       1   seq
......................................................................uUacuagagaguuuacucaucc...................   61      1   seq
......................................................................ugUcuagagaguuuacucaucc...................   40      1   seq
......................................................................ugacuagCgaguuuacucaucc...................   4       1   seq
......................................................................ugacuagagaguuuacucCucc...................   5       1   seq
......................................................................ugacuagagaguuuacucaucA...................   331     1   seq
......................................................................ugacuNgagaguuuacucaucc...................   4       1   seq
......................................................................ugacuagagUguuuacucaucc...................   28      1   seq
......................................................................ugacuagagaguuuacucaucU...................   3471    1   seq
......................................................................ugacCagagaguuuacucaucc...................   29      1   seq
......................................................................ugacuagagagGuuacucaucc...................   32      1   seq
......................................................................ugacuagagaguuuacuAaucc...................   99      1   seq
......................................................................ugacuagagaguAuacucaucc...................   95      1   seq
......................................................................ugacuGgagaguuuacucaucc...................   190     1   seq
......................................................................ugaAuagagaguuuacucaucc...................   54      1   seq
......................................................................ugacuagagagAuuacucaucc...................   79      1   seq
......................................................................ugacuagagaguuuacCcaucc...................   33      1   seq
......................................................................ugacuagagaguuuaUucaucc...................   16      1   seq
......................................................................ugaGuagagaguuuacucaucc...................   14      1   seq
......................................................................Ngacuagagaguuuacucaucc...................   117     1   seq
......................................................................ugacuCgagaguuuacucaucc...................   4       1   seq
......................................................................ugacuagagaguuuaGucaucc...................   15      1   seq
......................................................................ugacuagagaguuuacucauUc...................   73      1   seq
......................................................................ugacuagagCguuuacucaucc...................   4       1   seq
......................................................................ugacuagagaguCuacucaucc...................   34      1   seq
......................................................................ugacuagagaguuuUcucaucc...................   66      1   seq
......................................................................ugacuagaAaguuuacucaucc...................   143     1   seq
......................................................................ugacuagagaguGuacucaucc...................   27      1   seq
......................................................................ugacuUgagaguuuacucaucc...................   43      1   seq
......................................................................ugacuagagGguuuacucaucc...................   58      1   seq
......................................................................ugacuagagaguuuacucauAc...................   139     1   seq
......................................................................ugacuagagaguuuacucaCcc...................   36      1   seq
......................................................................ugacuagagaguuGacucaucc...................   6       1   seq
......................................................................ugacuagagaguuuacAcaucc...................   103     1   seq
......................................................................ugacuagagaguuuacucGucc...................   84      1   seq
......................................................................ugacuagagaguuuacGcaucc...................   7       1   seq
......................................................................ugaUuagagaguuuacucaucc...................   24      1   seq
......................................................................Agacuagagaguuuacucaucc...................   1227    1   seq
......................................................................ugacuagagaguuCacucaucc...................   75      1   seq
......................................................................ugacuagagaguuuaAucaucc...................   38      1   seq
......................................................................ugacuagagagCuuacucaucc...................   30      1   seq
......................................................................uNacuagagaguuuacucaucc...................   14      1   seq
......................................................................ugacGagagaguuuacucaucc...................   18      1   seq
......................................................................ugacuagagaguuuacuUaucc...................   220     1   seq
......................................................................ugacuagagaguuuacucaAcc...................   98      1   seq
......................................................................ugacuaAagaguuuacucaucc...................   174     1   seq
......................................................................ugacuagagaguuuacucUucc...................   32      1   seq
......................................................................ugacuaCagaguuuacucaucc...................   8       1   seq
......................................................................uCacuagagaguuuacucaucc...................   45      1   seq
......................................................................ugacuagagaguuuCcucaucc...................   6       1   seq
......................................................................ugacuagaUaguuuacucaucc...................   12      1   seq
......................................................................ugacuagagaguuuacucaucG...................   23      1   seq
......................................................................ugacuagagaguuuacucaucc...................   336399  0   seq
......................................................................ugGcuagagaguuuacucaucc...................   36      1   seq
......................................................................ugacuagagaguuuacucauGc...................   21      1   seq
......................................................................uAacuagagaguuuacucaucc...................   180     1   seq
......................................................................ugacuagUgaguuuacucaucc...................   37      1   seq
```

```
                      Star                                           Mature
uucucugcugacugccugucuguCUGGGUAAUCGCUCUAGACCAUGUGAUGUCGCAUGCUGCAGCGCUCAugacuagagaguuuacucauccagagaguagucuccuucuu
```

```
uucucugcugacugccugucugucuggguaaucgcucuagaccaugugaugucgcaugcugcagcgcucaugacuagagaguuuacucauccagagaguagucuccuucuu
......................................................................ugacuagagaguuuacucaGcc...................	13	1	seq
......................................................................Ggacuagagaguuuacucaucc...................	194	1	seq
......................................................................ugacuagagNguuuacucaucc...................	1	1	seq
......................................................................ugacuagagaguuuacuAaucca..................	2	1	seq
......................................................................ugacuagUgaguuuacucaucca..................	1	1	seq
......................................................................ugacuagagaguuuacucauccU..................	12878	1	seq
......................................................................ugacuagagaguuuacucauccG..................	34	1	seq
......................................................................Agacuagagaguuuacucaucca..................	1	1	seq
......................................................................ugacuagagaguuuacucauccN..................	1	1	seq
......................................................................ugacuagagaguuuacucauccC..................	33	1	seq
......................................................................ugacuagagaguuuUcucaucca..................	1	1	seq
......................................................................ugacuagaAaguuuacucaucca..................	1	1	seq
......................................................................ugacuagagaguuuacucaucca..................	814	0	seq
......................................................................ugacuagagaguuuacucGucca..................	1	1	seq
......................................................................ugacuagagaguuuacucaucAa..................	2	1	seq
......................................................................ugacuagagaguuuacucauGca..................	1	1	seq
......................................................................ugacuagagaguuuacucaucUa..................	4	1	seq
......................................................................ugacuagagagAuuacucaucca..................	1	1	seq
......................................................................Ngacuagagaguuuacucaucca..................	1	1	seq
......................................................................ugacuagagaguuuacucauAca..................	1	1	seq
......................................................................ugacuagagaguuuacAcaucca..................	1	1	seq
......................................................................ugacAagagaguuuacucaucca..................	1	1	seq
......................................................................ugacuagagaguuGacucaucca..................	1	1	seq
......................................................................ugacuagagaguuuacucauccaA.................	83	1	seq
......................................................................ugacuagagaguuuacucauccaU.................	8	1	seq
......................................................................ugacuagagaguuuacucauccaC.................	1	1	seq
......................................................................ugacuagagaguuuacucauccaAa................	128	1	seq
.......................................................................gacuagagaguuuacucau.....................	4	0	seq
.......................................................................gacuagagaguuuacucauc....................	208	0	seq
.......................................................................gaAuagagaguuuacucauc....................	1	1	seq
.......................................................................gacuagagaAuuuacucauc....................	1	1	seq
.......................................................................gacuagagaguuuacuUauc....................	1	1	seq
.......................................................................gacuagagaguuuacucaucG...................	1	1	seq
.......................................................................gacuagagaguuuacucaucA...................	2	1	seq
.......................................................................gacuaAagaguuuacucaucc...................	1	1	seq
.......................................................................gacuagagaguuuUcucaucc...................	1	1	seq
.......................................................................gacuagagaguuuaAucaucc...................	2	1	seq
.......................................................................gacuagagaguuCacucaucc...................	1	1	seq
.......................................................................gacuagagaguuuacucaAcc...................	1	1	seq
.......................................................................gacuagagaguuuacucauAc...................	7	1	seq
.......................................................................gacuagagaguuuacuAaucc...................	5	1	seq
.......................................................................gacuagagaguuuacucaucU...................	13	1	seq
.......................................................................gacuagaAaguuuacucaucc...................	2	1	seq
.......................................................................gacuagagagAuuacucaucc...................	1	1	seq
.......................................................................gacAagagaguuuacucaucc...................	8	1	seq
.......................................................................gacuGgagaguuuacucaucc...................	2	1	seq
.......................................................................gacuagagaguuuacAcaucc...................	3	1	seq
.......................................................................gacuaCagaguuuacucaucc...................	2	1	seq
.......................................................................gacuagagGguuuacucaucc...................	1	1	seq
.......................................................................gacuagagaguuuacucaucc...................	1978	0	seq
.......................................................................gGcuagagaguuuacucaucc...................	9	1	seq
.......................................................................gacuagagaguuuGcucaucc...................	2	1	seq
.......................................................................Uacuagagaguuuacucaucc...................	1	1	seq
.......................................................................gacuagagaguuuacucauccU..................	74	1	seq
.......................................................................gacuagagaguuuacucaucca..................	6	0	seq
.......................................................................gacuagagaguuuacucauccaAa................	1	1	seq
........................................................................acAagagaguuuacucaucc...................	1	1	seq
........................................................................acuagagaguuuacucaucc...................	2	0	seq
```
